# Acute Promyelocytic Leukemia: A Constellation of Molecular Events around a Single *PML-RARA* Fusion Gene

**DOI:** 10.3390/cancers12030624

**Published:** 2020-03-08

**Authors:** Alessandro Liquori, Mariam Ibañez, Claudia Sargas, Miguel Ángel Sanz, Eva Barragán, José Cervera

**Affiliations:** 1 Accredited Research Group in Hematology and Hemotherapy, Instituto de Investigación Sanitaria La Fe, 46026 Valencia, Spain; alessandro_liquori@iislafe.es (A.L.); claudia_sargas@iislafe.es (C.S.); 2 Department of Hematology, Hospital Universitario y Politécnico La Fe, 46026 Valencia, Spain; ibanyez_marcom@gva.es (M.I.); Miguel.Sanz@uv.es (M.Á.S.); barragan_eva@gva.es (E.B.); 3 Centro de Investigación Biomédica en Red de Cáncer (CIBERONC), 28029 Madrid, Spain

**Keywords:** acute promyelocytic leukemia, APL, NGS, minimal residual disease, MRD, PML-RARA, isoform, relapse, splicing

## Abstract

Although acute promyelocytic leukemia (APL) is one of the most characterized forms of acute myeloid leukemia (AML), the molecular mechanisms involved in the development and progression of this disease are still a matter of study. APL is defined by the *PML-RARA* rearrangement as a consequence of the translocation t(15;17)(q24;q21). However, this abnormality alone is not able to trigger the whole leukemic phenotype and secondary cooperating events might contribute to APL pathogenesis. Additional somatic mutations are known to occur recurrently in several genes, such as *FLT3*, *WT1*, *NRAS* and *KRAS*, whereas mutations in other common AML genes are rarely detected, resulting in a different molecular profile compared to other AML subtypes. How this mutational spectrum, including point mutations in the *PML-RARA* fusion gene, could contribute to the 10%–15% of relapsed or resistant APL patients is still unknown. Moreover, due to the uncertain impact of additional mutations on prognosis, the identification of the APL-specific genetic lesion is still the only method recommended in the routine evaluation/screening at diagnosis and for minimal residual disease (MRD) assessment. However, the gene expression profile of genes, such as *ID1, BAALC, ERG*, and *KMT2E,* once combined with the molecular events, might improve future prognostic models, allowing us to predict clinical outcomes and to categorize APL patients in different risk subsets, as recently reported. In this review, we will focus on the molecular characterization of APL patients at diagnosis, relapse and resistance, in both children and adults. We will also describe different standardized molecular approaches to study MRD, including those recently developed. Finally, we will discuss how novel molecular findings can improve the management of this disease.

## 1. Introduction

Acute promyelocytic leukemia (APL) is a biologically and clinically distinct subtype of acute myeloid leukemia (AML) with unique molecular pathogenesis, clinical manifestations and treatment that is cytogenetically characterized by a balanced translocation t(15;17) (q24;q21) [1,2,3]. This translocation involves the retinoic acid receptor alpha (*RARA*) gene on chromosome 17 and the promyelocytic leukemia (*PML*) gene on chromosome 15 that results in a *PML-RARA* fusion gene [4,5,6,7]. This fusion gene has been demonstrated to be responsible for cellular transformation, and confers a particular sensitivity to treatment with differentiating agents such as all-trans-retinoic acid (ATRA) plus chemotherapy or ATRA plus arsenic-trioxide (ATO), converting this once fatal leukemia into a highly curable disease both for pediatric and adult patients (cure rates of approximately 90%) [8,9,10,11]. The present review discusses some of the most recent findings concerning the molecular genetics of APL, beyond the *PML-RARA* fusion gene and its variants, both at diagnosis and relapse; and includes the main strategies for minimal residual disease (MRD) monitoring in patients. 

## 2. Pathophysiology of APL

The *PML-RARA* fusion gene is the most critical event involved in the pathogenesis of APL. This derives from a cytogenetic translocation leading to the rearrangement of *PML* and *RARA* genes [4,5,6,7]. 

**Figure 1:**
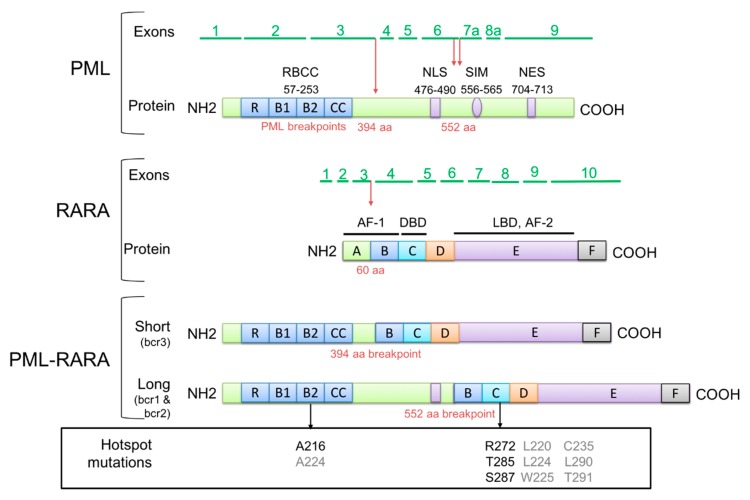
Structure of the acute promyelocytic leukemia (APL) primary event: promyelocytic leukemia (PML) and retinoic acid receptor-α (RARA) proteins and the corresponding PML–RARA fusion protein with the breakpoint regions (marked in red) and hotspot mutations (in the box at the bottom of the figure; in black are presented commonly mutated positions, and in grey rarer changes). In PML: RING finger (R), B boxes (B1 and B2), coiled-coil domain (CC), nuclear localization signal (NLS), SUMO-interacting motif (SIM), and nuclear export signal (NES). In RARA: N-terminal domain (A, B), including the activation function domain 1 (AF-1), DNA-binding domain (C), hormone-binding domain (E) and other regulatory domains (D and F).

*PML* is located in chromosome band 15q24, and contains nine exons producing several alternative spliced transcripts [12]. All the PML isoforms share the N-terminal region, harboring the RING-B-Box-Coiled-coil/tripartite motif (RBCC/TRIM) domain (encoded by exons 1 to 3); but differ either in the central (exons 4, 5 and 6) or in the C-terminal regions, due to alternative splicing (Figure 1). PML I, the longest one, which is distributed both in the nucleus and in the cytoplasm, is the only isoform containing a nuclear export signal (NES, exon 9) domain [12,13]. PML is mainly involved in tumor suppression and genomic instability [12,14,15,16], through it has constitutive or transient interactions with more than 170 proteins [17]. Most of these interactions are mediated either by the RBCC domain, which allows PML multimerization and organization in subnuclear structure, defined as nuclear bodies (NBs) [14,18,19]; or by other PML isoform-specific domains [20,21,22]. Therefore, through the creation of different binding interfaces, PML can be involved in several functional pathways, including p53-dependent and -independent apoptosis and senescence [23,24,25,26], stem cell self-renewal [16,27], epigenetic regulation and transcription of hematopoietic stem cells [20,21,28,29]. 

*RARA* is located in chromosome band 17q21, and comprises 10 exons encoding two isoforms, RARA1 and RARA2. Due to alternative promoter and exon usage, and alternative splicing, RARA isoforms differ from one another in the N-terminal Activation Function 1 (AF-1) domain (Figure 1) [30]. The RARA protein is a member of the nuclear receptor superfamily with high homology (90%) with RARB and RARG. This serves as a nuclear transcription factor when it is activated by retinoids, a class of molecules that are vitamers of vitamin A [31]. In the presence of the ligands, RARA forms heterodimers with retinoid X receptor (RXR) cofactor in order to bind specific *cis*-acting motifs (i.e., retinoic acid responsive elements, RARE), within the promoter region of the targeted gene (e.g., *RARA2*) [31,32]. However, in the absence of ligands, RARA/RXR dimers interact with nuclear corepressors, such as the silencing mediator of retinoid and thyroid hormone receptor (SMRT) and the nuclear receptor corepressor (N-CoR). The protein complexes thus formed cooperate with scaffolding proteins involved in the regulation of the gene transcription (i.e., Switch-Independent 3 A or B, Sin3A or Sin3B) and histone deacetylases (HDACs), resulting in nucleosome assembly and transcriptional repression [33]. Through this ligand presence-dependent switch, RARA acts as a differentiating agent of normal myeloid hematopoietic cells [34].

In APL, PML-RARA alters the nuclear structure of NBs, leading to their disruption into nuclear microspeckles [17]. This is likely due to the lack of the SUMO-binding motif within the PML moiety of the chimeric protein [14], and is a diagnostic hallmark of APL [17]. The PML-RARA oncogenic activity is exerted both through a dominant-negative and a gain-of-function effect [35,36]. This seems to be possible since PML-RARA fusion protein keeps the capacity to multimerize (conferred by the PML moiety) with several classes of protein, including the same PML, and other transcription and epigenetic factors [37,38,39]. On the one hand, PML-RARA produces a block of myeloid differentiation at the promyelocytic stage [40,41]. In this case, PML-RARA represses the transcription of several genes implicated in myeloid differentiation, such as those involved in the differentiation towards the granulocyte lineage, in a manner insensitive to physiological levels of retinoic ligands [42,43]. On the other hand, PML-RARA confers a survival and proliferative advantage to leukemic cells, resulting in the progressive accumulation of promyelocytes in the bone marrow of APL patients [36,44]. In this case, PML-RARA-RXR complexes mainly recognize atypical RAREs and interact with genomic regions characterized by a distinct pattern of chromatin modifications [39,45]. 

PML-RARA functions can be restored by the administration of two drugs, commonly used in clinical practice—ATRA and ATO [46,47]. These induce the PML-RARA degradation by binding to the RARA and PML parts of the fusion protein, respectively. Therefore, ATRA turns PML-RARA into a transcriptional activator, as a consequence of the release of several corepressors, including epigenetic enzymes (e.g., HDACs and DNA methyltransferases) and the interaction with a series of coactivators (e.g., coactivator-1, multi-protein complex including the cellular coactivator p300), leading to a more accessible chromatin [48]. On the other side, ATO induces different posttranscriptional modifications at the second B-box (B2) domain of the PML moiety, resulting in the change of the PML-RARA organization from microspeckles to NBs [47,49].

Mouse models have been used to elucidate most of the mechanisms described above and underlying the APL pathogenesis [50]. Transgenic and knock-in *PML-RARA* mice express a myeloproliferative disease phenotype and evolve to APL with a significant period of latency (6 to 18 months) and incomplete penetrance (15%–20% up to 90%, depending on the model) [40,41,51]. Nevertheless, these models have been a useful tool to investigate, among others, the oncogenic role of *PML-RARA* fusion and of its reciprocal [37,44,52], the co-existing events to the t(15;17) [53], the immune modulation of APL [54,55], and the mechanisms of response to therapy [56,57,58]. An alternative strategy for animal model production is to engraft human cells into immunodeficient mice strains [58,59,60]. Recently, Reinisch and colleagues demonstrated how to improve the engraftment of the xenotransplant by inducing the creation of a humanized bone marrow microenvironment. Remarkably, this approach allowed the researchers to identify the APL-initiating cells, associating them to committed myeloid CD34^−/lo^ GMP-like population [60].

## 3. *PML-RARA* Typical Isoforms

Four groups contributed to the identification of the chromosome breakpoints of the t(15;17) in the early 1990s [4,5,6,7]. These comprised three breakpoint clusters in three genomic regions (i.e., breakpoint cluster region, bcr) of the *PML* gene: in intron 6 (between exons 6 and 7), in exon 6, and in intron 3 (between exons 3 and 4), commonly known also as bcr1, bcr2 and bcr3, respectively (Figure 1) [61]. However, only one breakpoint in intron 2 has been identified in the *RARA* gene [61]. Depending on the breakpoint used and as a result of splicing, three different *PML-RARA* fusion transcripts can be generated, including the long (also known as L or bcr1 isoform), variant (V or bcr2), and short (S or bcr3) isoforms, respectively. In the case of bcr1 and bcr3 isoforms, exon 3 of *RARA* is spliced with exon 6 or 4 of *PML*, respectively [61]. These are the most common *PML-RARA* transcripts, identified in up to 90%–95% of patients. On the other hand, bcr2 isoform is produced from more complex splicing, resulting in the creation of a novel exon–exon junction between a cryptic donor splice site in *PML* exon 6 (GAAgtgagg, cDNA position 1685) and the acceptor splice site of exon 3 in *RARA* [62,63]. The same bcr have been reported in pediatric APL, with frequencies comparable with adults and mainly depending on the ethnic group [11,64]. Open reading frame is preserved in all cases, suggesting an oncogenic potential of the produced fusion proteins during APL onset and evolution.

Although all the patients harboring the t(15;17) express at least one of the *PML-RARA* isoforms, the reciprocal *RARA-PML* fusion is found only in 70% to 80% of them, either as *RARA1*- and *RARA2*-*PML* (breakpoints between exons 4 and 5), or only as *RARA1-PML* (breakpoints upstream of exon 4) [65,66]. Therefore, expression of the reciprocal chimeric protein might not be required for APL development. 

## 4. *PML-RARA* Atypical Isoforms

In addition to the typical *PML-RARA* isoforms, sporadic cases of t(15;17)-positive APL patients with atypical breakpoints, resulting in rare fusion transcripts, have been described (Table 1). Although the biological significance of many of these variants is still being debated, the analysis of their sequence is critical for understanding the causal molecular mechanisms and MRD monitoring [67]. Atypical bcr2, also defined as V-forms, are the most frequent variants [61,62,63,68,69]. These are characterized by cDNA deletions of either the distal region of *PML* exon 6 (from 8 to 146 nucleotides), or the entire exon 6, as a result of a mis-splicing event or a genomic break within *PML* exon 6 (rarely in *PML* exon 5). Likewise, atypical bcr2 are frequently associated with insertions of three to 127 extra nucleotides (1 to 42 extra amino acids) of genomic DNA from *RARA* intron 2 [63,68,69,70,71,72,73,74]. In most of the cases, V-forms are “private”, being observed in single APL patients. However, a few cases share the activation of a novel donor splice site in *PML* exon 6 [72,73], or express a minor *PML-RARA* transcript with exon 5 skipped [72,74]. 

Atypical cases of bcr1 with breakpoints located downstream of *PML* intron 6 have also been reported. In these cases, the donor splice site of exon 7a or 7b is spliced directly with *RARA* exon 3 [70,77,79], or indirectly through the insertion of genomic DNA (19 to 119 nucleotides) from *PML* intron 7 or *RARA* intron 2, respectively [67,74,78,80]. Notably, Yi and colleagues reported a case in which a reverse sequence of 19 nucleotides originated from *PML* exon 7c complementary sequence was inserted between part of exon 7c (308 nucleotides) and *RARA* exon 3 [80]. In addition, partial deletions of *RARA* exon 3 have also been observed both in atypical bcr1 as well as in bcr3 transcripts [67,73,79]. These rare bcr3 isoforms originate from breakpoints located downstream of *PML* exon 4, which is then commonly involved in the splicing with *RARA* exon 3 [73,74,81,82,83]. In this case, as for bcr2 and bcr1 variants, insertions of genomic DNA sequences might also occur at the junction between *PML* and *RARA* genes [74,82,83]. Among others, a short insertion of nine nucleotides (CCCCCAGTT) of unknown origin has been reported in a *PML-RARA* fusion between *PML* exon 4 and part of the exon 1 of *RARA2*. This was the first time that a fusion transcript involving the alternative isoform of *RARA* was found in an APL patient [82]. 

## 5. Responsiveness to Treatment of APL Patients Depending on *PML-RARA* Isoforms

Both typical and atypical *PML-RARA* isoforms have been correlated with diverse prognosis and responsiveness to treatments (e.g., ATRA), likely due to the different PML domains retained in the fusion protein. However, the results from the different studies are controversial, mainly because of the substantial clinical heterogeneity associated. Some reports have shown that bcr3 patients would have a poor prognosis and an aggressive disease course [84], as well as patients harboring *PML* breakpoints downstream of intron 6 [67,70,75,77]. However, other studies have described cases with a good prognosis, or at least similar to that of APL patients with typical transcripts, even in the presence of atypical isoforms [67,72,78,80]. Therefore, according to the most recent recommendations, standard therapy should not be changed based on the *PML-RARA* isoforms [85]. 

## 6. APL Molecular Variants

In roughly 1% to 2% of APL patients, novel translocations other than t(15;17) at either the cytogenetic or molecular level have been identified. To date, 12 molecular fusion variants of APL have been described, all involving the *RARA* gene [86] (Table 2). The *ZBTB16* (formerly *PLZF*)*-RARA* fusion, derived from the t(11;17)(q23;q21) rearrangement [87], has been reported in more than 30 patients, making it the most frequent APL molecular variant [85,86]. Other reported translocations led to the rearrangement of *RARA* gene with *NPM1* [88], *NUMA1* [89], *STAT5B* [90], *PRKAR1A* [91], *FIP1L1* [92], *BCOR* [93], *NABP1* (formerly *OBFC2A*) [94], *TBL1XR1* (formerly *TBLR1*) [95], *GTF2I* [96], *IRF2BP2* [97], and *FNDC3B* [98]. Although the detection of these APL molecular variants can escape standard molecular diagnosis, their characterization is essential for the appropriate management and treatment of patients, since a variable sensitivity to treatment has been reported [85] (Table 2).

In addition, two AML resembling APL cases with no *RARA* gene implication have been reported both involving the retinoid acid receptor gamma gene (*RARG*, 12q13.13). In the first case, *RARG* was fused to the nucleoporin 98 (*NUP98*) gene to produce the *NUP98-RARG* transcript [99]. In the second case, *RARG* was fused to the *PML* gene in a patient with the hypergranular subtype of APL [100]. Due to the early discontinuation of ATRA in both patients, the sensitivity to ATRA treatment was not certain in these cases, although in vitro studies suggest that at least *NUP98-RARG* rearrangement might be resistant to this drug [101].

## 7. Additional Molecular Events to *PML-RARA*

Although *PML-RARA* rearrangement is the cytogenetic hallmark of APL, in vitro studies performed on transgenic mice support the hypothesis that secondary cooperating genetic events accumulated over time are essential to ultimately trigger the whole leukemic phenotype [113,114]. A wide number of genomic alterations have been associated with APL, but only some of them occur recurrently [115,116,117]. This links with the peculiar morphology and distinctive clinical features compared with other forms of AML.

### 7.1. Additional Chromosomal Abnormalities

Almost half of pediatric and adult APL patients harbor further chromosomal alterations (ACAs) in addition to the t(15;17) identified by conventional cytogenetics [11,118]. As in other AML subtypes [119], deletion 7q and trisomy 8 are the most prevalent alterations. The gain of additional material by chromosome 8 leads to *MYC* deregulation in APL cells [120] that can act in cooperation with the *PML-RARA* fusion gene [121,122,123]. Other alterations have also been identified, but less frequently and in a non-recurrent manner (≤3%) [118,124,125]. With the probable exception of complex karyotypes with three or more ACAs [126], it is accepted that ACAs do not affect the prognosis of patients with APL. 

In addition, studies carried out by single-nucleotide polymorphism array (SNP-A) tools have shown that acquired non-recurrent cytogenetic cryptic (i.e., submicroscopic) variations are relatively common and impact negatively on the outcome of APL patients [124,125]. However, most of these studies lack paired germinal samples, sparking controversy on those copy number alterations not reported in public databases and thus categorized as acquired variations. By contrast, studies carried out with a matched germline sample have revealed a mean of 0.93 cryptic lesions per case, suggesting that few additional aberrations are needed in this type of leukemias [125], in contrast to multiple micro-deletions that are usually found in other types of leukemias, such as acute lymphoblastic leukemia [127]. 

### 7.2. Gene Mutations at Diagnosis, Relapse and Resistance

**Figure 2:**
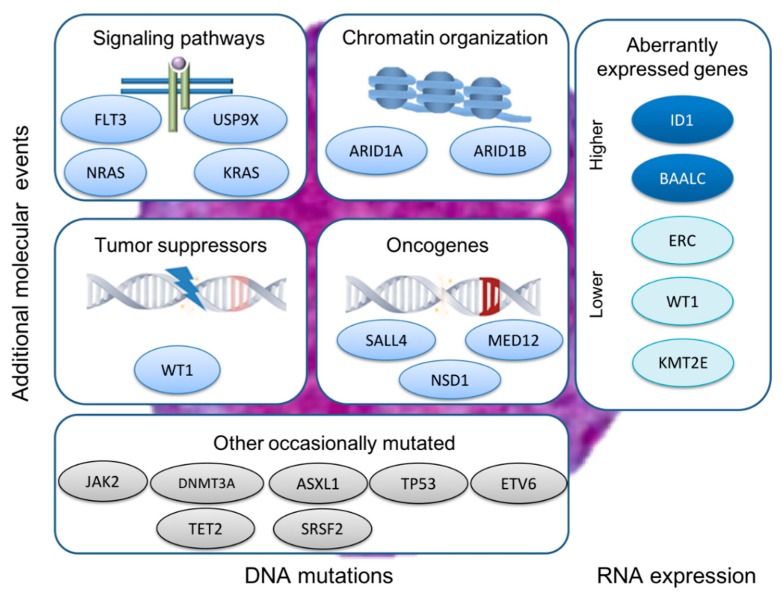
Acute promyelocytic leukemia (APL) molecular profile. APL and associated molecular events categorized by oncogenic mechanism. DNA mutations in genes involved with signaling pathways, chromatin organization, tumor suppressor and oncogenes, among others, and aberrantly expressed genes associated with *PML-RARA* rearrangement (in blue commonly mutated; in grey occasionally mutated).

The genetic features of APL have been analyzed by next-generation sequencing (NGS) approaches, showing a mutational landscape different from other myeloid malignancies [115,116,128] (Figure 2). This molecular profile is defined by recurrent alterations in genes associated with signaling pathways (*FLT3, NRAS, KRAS*), tumor suppression (*WT1*), chromatin organization (*ARID1B* and *ARID1A*), oncogenes (*SALL4, MED12, NSD1*), and rarer mutations in other AML-pathways, including *NPM1* mutations, DNA methylation (*DNMT3A, IDH1/2* and *TET2),* or epigenetic regulation (*ASXL1*) [115,116,123,128]. Although mutations in epigenetic modifier genes (i.e., *DNMT3A, TET2, IDH1, IDH2*, and *ASXL1*) jointly represent less than 6% of APL cases, these have been associated with a poor prognosis with regard to overall survival and disease free survival [129].

*FLT3*-ITD and *FLT3*-D835 mutations in the *FLT3* gene are the most frequent co-occurring events to *PML-RARA* both in pediatric and adult APL, representing up to 40% of cases, mainly associated with elevated white blood cell (WBC) counts [11,130]. However, the prognostic implication of these mutations remains controversial. While a recent meta-analysis found that *FLT3* mutations are associated with elevated WBC counts and poorer clinical outcomes [130], in the largest study reported so far, we were unable to demonstrate an independent prognostic value of these mutations when WBC count was included in multivariable analysis [131]. It has been found that alterations in *FLT3*-ITD impedes retinoic acid, but no arsenic, responses in a murine model [132], and that ATRA and ATO selectively exert synergistic cytotoxicity against *FLT3*-ITD AML cells via co-inhibition of FLT3 signaling pathway [133,134]. Once in the clinical arena, results from a small series of low-to-intermediate risk patients with APL suggest the antileukemic advantage of ATRA plus ATO in patients with *FLT3*-ITD [133,134]. Nevertheless, these studies have been considered not evidence-based enough to recommend any *FLT3*-oriented therapy in APL out of clinical trials [85]. 

In addition to *FLT3*, the profile of mutations in pediatric APL is similar to adult patients, where recurrent mutations in *WT1, USP9X, NRAS*, and *ARID1A* are strongly related to de novo APL and in *WT1* with relapsed APL [11,64]. Likewise, additional mutations have been detected in a non-recurrent manner, affecting, mainly, MAPK pathway (*BRAF, KIT, PDGFRA*) or transcriptional regulation (*MED12, KDM6A*) in both, adult and pediatric patients [64,135]. Underlying the heterogeneity of genes implicated in the disease, in silico strategies, based on network enrichment analysis, have identified aberrant gene interactions within pathways potentially implicated in APL leukemogenesis [128]. Therefore, related biological functions may have a similar effect on leukemogenesis, requiring a few concerted molecular events to develop the APL phenotype. Therefore, the alteration of key cellular pathways caused by diverse but not necessarily frequent or recurrent mutations could promote the arising and maintenance of APL [115,116,123,128]. However, further analyses are required to confirm this assumption. 

At diagnosis, about 70% of APL patients harbor a mean of 0.96 somatic mutations (range 0–2) additionally to *PML-RARA* rearrangement [115,116,123,128,135]. However, this average is greater at relapse, with a mean of three additional somatic mutations (range 0–61), mostly acquired through the disease course [115,116,123,128,135]. These mutations are mainly localized in *NRAS, RUNX1* and *ARID1B*, and rarely found in newly diagnosed APL, suggesting their possible role as predictive markers of relapse [136,137]. More recently, point mutations in the *PML-RARA* fusion gene have been described in up to 30% of the relapsed cases [136] (Figure 1). These studies suggested that mutations occurred in the two moieties of *PML-RARA* could contribute to 10–15% of patients who relapse or those eventually resistant to treatment by targeted therapy with ATRA and ATO [136,138]. *PML* mutations are thought to inhibit ATO binding whereas *RARA* mutations could reduce the affinity to ATRA binding [136,138]. In many cases, these mutations in *PML* or *RARA* are already present in a low burden at diagnosis, as unveiled by NGS, and expanded under treatment selection during disease evolution, acquiring, occasionally, mutations in other genes [136]. Therefore, the early finding of these mutations emerges as a possible future approach to modify therapeutic strategies. All the described mutations are located in critical domains identified as hotspots in PML (A216) and RARA (R272, T285 and S287) (Figure 1) [138,139], and appear to be mutually exclusive with *FLT3* mutations, suggesting their different role in leukemogenesis and disease progression. Additional novel alterations have been described in both PML (A224G) and RARA (L220P, L224I, W225C, C235F, L290V, and T291A) moieties. Moreover, concomitant mutation patterns have been identified in a non-recurrent manner during the evolution of the disease, affecting genes involved in pathways associated with clonal hematopoietic expansion, such as signaling pathways (*JAK2)*, DNA methylation (*DNMT3A* and *TET2*), epigenetic regulation (*ASXL1*), splicing (*SRSF2*), transcriptional factors (*ETV6*), and tumor suppression (*TP53*) [136]. In addition, mutations present at diagnosis in *WT1*, epigenetic or kinase factor genes would be retained in the relapse, promoting the acquisition of additional aberrations, while in *NRAS* or *FLT3* they would disappear, allowing access to new molecular subclones involved in the relapse [135,137]. Accordingly, two different models of disease progression have been suggested: model 1, in which relapse may originate from the persistence of the dominant founder clone, present at diagnosis, which survived and evolved into the relapse clone, expanding with an unclear mechanism; and model 2, where mutated subclones arise under selective pressure of treatment and with the acquisition of novel mutations, which resulted in the clonal expansion of the affected cells [136]. In model 1, patients might have oncogenic alterations and/or mutations, either inherited or acquired, that conferred a resistance mechanism to treatment. In model 2, the founder clone was displaced at relapse by new subclones, probably due to selective pressure through competition between subclones or as a consequence of the chemotherapy. In this second model, where subclones are totally different from the diagnostic clones, is especially relevant the development of new management strategies that allow us to detect those cases in which the treatment will fail [136,140]. Ongoing studies focused on clonal evolution and resistance suggest the use of new retinoid molecules, together with ATRA or ATO treatments, with novel therapeutic targets acting through alternative molecular mechanisms [129,140]. However, further investigation in several clinical trial settings is warranted.

Together with somatic mutations, aberrantly expressed genes have been identified in APL patients. For example, a recent study has revealed that the down-regulation of *IRF8* driven by PML-RARA could trigger the advent and preservation of the leukemic clone in a mouse model, contributing to the acquisition of cooperative genomic events [42]. In addition, the expression profile of genes such as *ID1, BAALC, ERC, WT1* and *KMT2E,* alongside additional molecular events (such *FLT3*-ITD status and ∆Np73/TAp73 expression ratio), could improve the treatment outcome prediction in high-risk APL patients (Figure 2). Consequently, an integrative score in APL (ISAPL), combining gene mutations with expression analysis, has been proposed, categorizing patients in two different sets with significant differences in remission rate, relapse and survival [141]. The ISAPL could assist in more narrowly monitoring the measurable MRD and design of improved treatment schemes. 

The gene expression pattern of APL has also been investigated with an elegant chemo-genomic strategy to identify novel molecular targets and drugs for the treatment of patients. By comparing the transcriptional profiles of APL cases with those induced by gene mutations or drugs, Chen and collaborators identified a list of at least 15 proteins (i.e., PML, RARA, ABL1, AFF1, BCR, CEBPA, FGFR1, FOS, HDAC3, MEIS1, NPM1, NUP98, PDGFRB, PTEN, and SPI1) potentially able to revert the transcriptional patterns of APL when properly targeted with specific drugs [142].

## 8. Standardized Molecular Approaches to Study Minimal Residual Disease 

Although roughly 90% of newly diagnosed APL patients achieve long-term remission having received only targeted therapy, a considerable proportion of those patients relapsed with no evidence of predictive clinical parameters [115,116,123,128]. As a target for MRD, the ready detection of *PML-RARA* transcript at the post-consolidation phase by modern PCR-based techniques could improve outcome prediction through the rapid accurate assessment of the response to treatment [143,144]. Several retrospective studies have pointed out that molecular relapse is an independent prognostic factor which precedes the reappearance of APL blasts [84,145,146,147,148,149,150]. Successive studies established that negative longitudinal PCR assays performed on bone marrow after completing therapy are strongly related with long-term remission. In addition, molecular assessment previous to stem cell transplantation predicts the risk of relapse after stem cell engraftment [150,151,152,153,154,155,156]. Consequently, several expert panels, including the European Leukemia Net and the US international working Group with the NCCN, recommended in the 2003 Guidelines the establishment of molecular response, defined as undetectable *PML-RARA* transcript by PCR test with sensitivity of 10^−4^ [157], as a valuable end point during the follow-up. Thus, the eventual detection of molecular relapse in early stages allows treatment intervention that could improve the outcome of these patients [158]. 

Initial studies analyzed the presence of *PML-RARA* transcript after treatment by nested RT-PCR [159,160,161]. However, real time quantitative approaches (qRT-PCR), which afford comparable sensitivity but are more readily standardized, are the current gold standard strategy for molecular monitoring in APL [162]. A number of large studies have proved that the most important MRD end point in APL treated with ATRA- or ATO-based therapies is the achievement of PCR negativity for *PML-RARA* at the end of consolidation therapy [10,134,144,163,164,165,166,167]. However, it is controversial whether serial PCR measurements of *PML-RARA* during treatment are of value outside of clinical trials [168]. Recently, the MRD consensus document of the European Leukemia Net recommended not changing the treatment plan for an individual with detectable levels of *PML-RARA* before the end of consolidation [169]. Furthermore, given the low rates of relapse observed in patients presenting with low and intermediate risk of disease (by Sanz score) [170], MRD analysis in these patients should be performed in bone marrow until the patient achieves MRD negativity and then should be terminated. Only patients with high risk APL should continue sequential MRD monitoring after completion of therapy for at least 2 years [169]. 

Although quantification using peripheral blood is an attractive option, a study comparing both sources showed that conversion to PCR positivity was detected earlier in bone marrow than in peripheral blood [144]. However, the main limitation is that the qRT-PCR technique provides unreliable results when the tumor burden is very low (within the threshold of sensitivity). The need for more sensitive PCR techniques is driven to improve clinical decision-making. A new alternative method in monitoring MRD is droplet digital PCR (ddPCR), which provides an accurate and highly sensitive absolute quantification of the *PML-RARA* transcript, including atypical *PML*-*RARA* fusion transcripts [74,171]. Using this new method, residual transcripts could be detected independently of the kinetics of molecular relapse, which is APL transcript-specific and not time-dependent [171]. For instance, this technique could be a feasible and valuable tool in order to improve risk stratification at diagnosis and complement MRD monitoring, especially for patients scored as to be at high risk of relapse. Nevertheless, further studies should be performed to validate and standardize this method for MRD monitoring in APL.

## 9. Conclusions 

In an era of medicine in which several novel cases of targeted therapy are emerging for AML (e.g., Midostaurin for *FLT3*mut, and Enasidenib for *IDH*mut), APL has been a pioneer, considering that this has been a reality for this neoplasm for 30 years [8,172]. It should be noted that the introduction of ATRA and ATO into clinical practice has been accompanied by a huge number of studies at molecular level, which we have tried to revise to the best of our knowledge, to understand the pathophysiology of the disease and its responsiveness to treatment. However, many aspects remain to be further studied, since early death rate still reaches 15%, and up to 10% of patients relapse or become resistant. As we described in this review, genomics has made it possible to widen the APL genetic landscape. However, it should be through the integration of genomics with other -*omic* science (i.e., epigenomics, transcriptomics and metabolomics) that we might broaden our perspective and explain the hitherto unsolved issues. This represents a great challenge for researchers in the near future, but some of the pillars seem to have already been built with the proposal, among others, of the first integrative score [141].

## Figures and Tables

**Table 1:** Molecular features of typical and atypical *PML-RARA* isoforms.

Type, Reported Cases	*PML*, Breakpoint	*PML*, Splice Site	Genomic Insertions	*RARA*, Breakpoint	*RARA*, Splice Site	Reference
Typical isoforms						
Bcr1 (58–75% of pts)	Intron 6	CAGgtaggg		Intron 2	ctctagCCA	[61,75,76]
Bcr2 (5–10% of pts)	Exon 6	GAAgtgagg		Intron 2	ctctagCCA	[61,75,76]
Bcr3 (15–33% of pts)	Intron 3	CAGgtgagt		Intron 2	ctctagCCA	[61,75,76]
Atypical isoforms						
Bcr1						
2 pts	Exon 7a	TCGgtgagt		Intron 2	ctctagCCA	[70,77]
1 pt	Exon 7a	TGGtgatca	T + chr17:12049-12168 (119 nt)	Intron 2	ctctagCCA	[74]
1 pt	Exon 7a	CAGctcgga	chr17: 40342767-40342865 (100 nt)	Intron 2	ctctagCCA	[78]
1 pt	Exon 7a	TCGgtgagt	chr15:74036990-74037095 (106 nt) + ATCT	Exon 3	cagcccTCC	[67]
1 pt	Exon 7b	GGAtccgct		Intron 2	ctctagCCA	[77]
1 pt	Exon 7b	CGCcttcgc		Exon 3	agcagcAGT	[79]
1 pt	Exon 7c	GATcgctgg	tctgtgctctgtacaacag (19 nt, reverse inserted sequence originated from *PML* Exon 7c complementary sequence)	Intron 2	ctctagCCA	[80]
Bcr2						
2 pts	Exon 6	GAGctcccc		Intron 2	ctctagCCA	[72,73]
1 pt	Exon 6	GCCagtggc	chr17: 40338105-40338139 (35 nt)	Intron 2	ctctagCCA	[70]
1 pt	Exon 6	GGCaaggtt	ccttg (5 nt from *RARA*)	Intron 2	ctctagCCA	[71]
1 pt	Exon 6	GGAggggaa	chr17: 15582-15596 (15 nt)	Intron 2	ctctagCCA	[74]
1 pt	Exon 6	CCGgagcag	aagcccgtcttccttttag (19 nt from *RARA*)	Intron 2	ctctagCCA	[69]
1 pt	Exon 6	GAGctcccc	gagtccttctgcaggaagaggagagattg (29 nt from *RARA*)	Intron 2	ctctagCCA	[63]
1 pt	Exon 6	TCCccggag	tcccctcttctctctctag (19 nt from *RARA*)	Intron 2	ctctagCCA	
1 pt	Exon 6	CTAgcccca	tggacacacaggttggag (18 nt from *RARA*)	Intron 2	ctctagCCA	
1 pt	Exon 6	TAGccccag	tcttagag (8 nt from *RARA*)	Intron 2	ctctagCCA	
1 pt	Exon 6	GTCatagga	chr17:40343445-40343571 (127 nt)	Intron 2	ctctagCCA	
1 pt	Exon 6	GAAgtgagg		Intron 2	ctctagCCA	
1 pt	Exon 6	CCCaacagc	gaaggactggacacacaggttggag (25 nt from *RARA*)	Intron 2	ctctagCCA	
1 pt	Exon 6	GCAaccacg	gag	Intron 2	ctctagCCA	
1 pt	Exon 6	AACcacgtg	gcccggcacacatacaat (18 nt from *RARA*)	Intron 2	ctctagCCA	
1 pt	Exon 6	ACGtggcca	gagcca	Intron 2	ctctagCCA	
1 pt	Exon 6	CGTggccag	actctttcttagag (14 nt from *RARA*)	Intron 2	ctctagCCA	
1 pt	Exon 6	TGGccagtg	gag	Intron 2	ctctagCCA	
1 pt	Exon 6	GCGccgggg		Intron 2	ctctagCCA	
1 pt	Exon 6	GCCggggag	chr17:40342828-40342867 (40 nt)	Intron 2	ctctagCCA	
1 pt	Exon 6	CCGgggagg	agtttggg (8 nt from *RARA*)	Intron 2	ctctagCCA	
Bcr3						
2 pts	Intron 4	CTGgtgaga		Intron 2	ctctagCCA	[73,74]
1 pt	Intron 4	unknown		Intron 2	unknown	[81]
1 pt	Intron 4	CTGgtgaga	chr15:74050024-74050143 (120 nt)	Intron 2	ctctagCCA	[74]
1 pt	Intron 4	CTGgtgaga	cccccagtt	Exon 1 of *RARA2*	catctgCAG	[82]
1 pt	Intron 4	CTGgtgaga	chr17:40343186-40343225 (40 nt)	Intron 2	ctctagCCA	[83]

Reported isoforms have been compared with the reference sequences of *PML* and *RARA* (GenBank accession numbers: NM_033238.2; NM_000964.3). “*PML* and *RARA* splice site” columns show the nucleotides involved in exon–exon boundaries between *PML* and *RARA* (upper cases) genes, as well as genomic nucleotides that are spliced-out (lower cases) from the fusion transcripts. In addition, inserted sequences are shown in uppercase when they were of unknown origin and in lowercase when it was possible to define their origin. Pt, patient. Nt, nucleotides.

**Table 2:** APL molecular variants.

APL molecular Variants	Translocations	ATRA Sensitivity	ATO Sensitivity	No. of Cases Reported	Gene Other Than *PML*, Breakpoint	Gene Other Than *PML*, Splice Site	Genomic Insertions	*RARA*, Breakpoint	*RARA*, Splice Site	Reference
*ZBTB16 (PLZF)-RARA*	t(11;17) (q23;q21)	Poorly responsive	Poorly responsive	>30 [85]	Intron 3	CAGgtaggc		Intron 2	ctctagCCA	[102]
					Intron 4	CTGgtgagt		Intron 2	ctctagCCA	[102]
*NPM1-RARA*	t(5;17) (q35;q21)	Sensitive	ND	5 [103]	Intron 5	CAGgtagag		Intron 2	ctctagCCA	[103]
					Intron 5	CAGgtagag	79 nt with no homology to sequences in the GenBank or EMBL databases	Intron 2	ctctagCCA	[104]
					Intron 4	TAGgtatgt		Intron 2	ctctagCCA	[103]
*NUMA1 (NUMA)-RARA*	t(11;17) (q13;q21)	Sensitive	ND	1	Intron 23	CAGgtgagg		Intron 2	ctctagCCA	[89]
*STAT5B-RARA*	der(17)	Poorly responsive	Poorly responsive	11 [105]	Intron 15	CTCgtgagt		Intron 2	ctctagCCA	[90,105]
*PRKAR1A-RARA*	t(17;17) (q21;q24) or del(17) (q21;q24)	Sensitive	Sensitive	1	Intron 2	AAGgtaaaa		Intron 2	ctctagCCA	[91]
*FIP1L1-RARA*	t(4;17) (q12;q21)	Sensitive in 1 case	ND	2	Intron 15	ATGgtaagt		Intron 2	ctctagCCA	[92]
					Intron 13	CGGgtaaat		Intron 2	ctctagCCA	[106]
*BCOR-RARA*	t(X;17) (p11;q21)	Sensitive in 2 cases	Insensitive in 1 case	2	Intron 12	CAGgtatga		Intron 2	ctctagCCA	[93]
					Exon 12	CAGgtagaa		Intron 2	ctctagCCA	[107]
*NABP1 (OBFC2A) -RARA*	t(2;17) (q32;q21)	Sensitive in vitro	ND	1	Intron 5	TGGgtaaga		Intron 2	ctctagCCA	[94]
*TBL1XR1 (TBLR1)-RARA*	t(3;17) (q26;q21)	Insensitive	ND	4 [108]	Intron 5	CAAgtgagc		Intron 2	ctctagCCA	[95]
					Intron 5	CAAgtgagc	*	Intron 2	ctctagCCA	[108]
*GTF2I-RARA*	t(7;17) (q11;q21)	Sensitive	Sensitive	1	Intron 6	TAGgtaagt		Intron 2	ctctagCCA	[96]
*IRF2BP2-RARA*	t(1;17) (q42;q21)	Sensitive	Sensitive	5 [109]	Exon 2	TGTcccctg		Intron 2	ctctagCCA	[97,109]
					Exon 1	AAGgtgcgg		Intron 2	ctctagCCA	[110]
					Intron 1	CAGgtaggg		Intron 2	ctctagCCA	[111]
					Exon 1	CAGgcaggt		Intron 2	ctctagCCA	[111,112]
*FNDC3B-RARA*	t(1;17) (q42;q21)	Sensitive	Sensitive	1	Intron 24	AAGgtgtgt		Intron 2	ctctagCCA	[98]

Genes involved in APL molecular variants are reported according to the HUGO Gene Nomenclature Committee (https://www.genenames.org/). Previous symbols are reported in brackets. * According to molecular analysis, *RARA* on chromosome 17 had a 12-kbp deletion, of which a small region was inserted into chromosome X and the major part was inserted within *TBL1XR1* gene between exons 5 and 6 on chromosome 3 [108].
