# Correction: Liquori et al. Acute Promyelocytic Leukemia: A Constellation of Molecular Events around a Single PML-RARA Fusion Gene. *Cancers* 2020, *12*, 624

**DOI:** 10.3390/cancers13143440

**Published:** 2021-07-09

**Authors:** Alessandro Liquori, Mariam Ibañez, Claudia Sargas, Miguel Ángel Sanz, Eva Barragán, José Cervera

**Affiliations:** 1 Accredited Research Group in Hematology and Hemotherapy, Instituto de Investigación Sanitaria La Fe, 46026 Valencia, Spain; alessandro_liquori@iislafe.es (A.L.); claudia_sargas@iislafe.es (C.S.); 2 Department of Hematology, Hospital Universitario y Politécnico La Fe, 46026 Valencia, Spain; ibanyez_marcom@gva.es (M.I.); Miguel.Sanz@uv.es (M.Á.S.); barragan_eva@gva.es (E.B.); 3 Centro de Investigación Biomédica en Red de Cáncer (CIBERONC), 28029 Madrid, Spain

The authors wish to make the following corrections to this paper [1]:

In the last row of Table 2, translocation is t(3;17)(q26;q21) instead of t(1;17)(q42;q21).

In the last row of Table 2, ATO sensitivity should be changed to ND (not determined) instead of “Sensitive”.

The new Table 2 is shown below:

**Table 2:** APL molecular variants.

APL Molecular Variants	Translocations	ATRA Sensitivity	ATO Sensitivity	No. of Cases Reported	Gene Other Than *PML*, Breakpoint	Gene Other Than *PML*, Splice Site	Genomic Insertions	*RARA*, Breakpoint	*RARA*, Splice Site	Reference
*ZBTB16 (PLZF)-RARA*	t(11;17)(q23;q21)	Poorly responsive	Poorly responsive	>30 [85]	Intron 3	CAGgtaggc		Intron 2	ctctagCCA	[102]
					Intron 4	CTGgtgagt		Intron 2	ctctagCCA	[102]
*NPM1-RARA*	t(5;17)(q35;q21)	Sensitive	ND	5 [103]	Intron 5	CAGgtagag		Intron 2	ctctagCCA	[103]
					Intron 5	CAGgtagag	79 nt with no homology to sequences in the GenBank or EMBL databases	Intron 2	ctctagCCA	[104]
					Intron 4	TAGgtatgt		Intron 2	ctctagCCA	[103]
*NUMA1 (NUMA)-RARA*	t(11;17)(q13;q21)	Sensitive	ND	1	Intron 23	CAGgtgagg		Intron 2	ctctagCCA	[89]
*STAT5B-RARA*	der(17)	Poorly responsive	Poorly responsive	11 [105]	Intron 15	CTCgtgagt		Intron 2	ctctagCCA	[90,105]
*PRKAR1A-RARA*	t(17;17)(q21;q24) or del(17)(q21;q24)	Sensitive	Sensitive	1	Intron 2	AAGgtaaaa		Intron 2	ctctagCCA	[91]
*FIP1L1-RARA*	t(4;17)(q12;q21)	Sensitive in 1 case	ND	2	Intron 15	ATGgtaagt		Intron 2	ctctagCCA	[92]
					Intron 13	CGGgtaaat		Intron 2	ctctagCCA	[106]
*BCOR-RARA*	t(X;17)(p11;q21)	Sensitive in 2 cases	Insensitive in 1 case	2	Intron 12	CAGgtatga		Intron 2	ctctagCCA	[93]
					Exon 12	CAGgtagaa		Intron 2	ctctagCCA	[107]
*NABP1 (OBFC2A)-RARA*	t(2;17)(q32;q21)	Sensitive in vitro	ND	1	Intron 5	TGGgtaaga		Intron 2	ctctagCCA	[94]
*TBL1XR1 (TBLR1)-RARA*	t(3;17)(q26;q21)	Insensitive	ND	4 [108]	Intron 5	CAAgtgagc		Intron 2	ctctagCCA	[95]
					Intron 5	CAAgtgagc	*	Intron 2	ctctagCCA	[108]
*GTF2I-RARA*	t(7;17)(q11;q21)	Sensitive	Sensitive	1	Intron 6	TAGgtaagt		Intron 2	ctctagCCA	[96]
*IRF2BP2-RARA*	t(1;17)(q42;q21)	Sensitive	Sensitive	5 [109]	Exon 2	TGTcccctg		Intron 2	ctctagCCA	[97,109]
					Exon 1	AAGgtgcgg		Intron 2	ctctagCCA	[110]
					Intron 1	CAGgtaggg		Intron 2	ctctagCCA	[111]
					Exon 1	CAGgcaggt		Intron 2	ctctagCCA	[111,112]
*FNDC3B-RARA*	t(3;17)(q26;q21)	Sensitive	ND	1	Intron 24	AAGgtgtgt		Intron 2	ctctagCCA	[98]

The genes involved in APL molecular variants are reported according to the HUGO Gene Nomenclature Committee (https://www.genenames.org/, accessed on 8 March 2020). Previous symbols are reported in brackets. * According to molecular analysis, *RARA* on chromosome 17 had a 12-kbp deletion, of which a small region was inserted into chromosome X and the major part was inserted within the *TBL1XR1* gene between exons 5 and 6 on chromosome 3 [108].

The authors would like to apologize for any inconvenience caused to the readers by these changes. The original manuscript has been updated.
